# Assessing the potential utility of large language models for assisting community health workers: protocol for a prospective, observational study in Rwanda

**DOI:** 10.1136/bmjopen-2025-110927

**Published:** 2025-10-14

**Authors:** Vaishnavi Menon, Natnael Shimelash, Samuel Rutunda, Cyprien Nshimiyimana, Lucinda Archer, Mira Emmanuel-Fabula, Derbew Fikadu Berhe, Jaspret Gill, Emery Hezagira, Eric Remera, Richard Riley, Rex Wong, Alastair K Denniston, Bilal Akhter Mateen, Xiaoxuan Liu

**Affiliations:** 1 University of Birmingham, Birmingham, UK; 2 University of Global Health Equity, Kigali, Rwanda; 3 Digital Umuganda, Kigali, Rwanda; 4 Centre for the Fourth Industrial Revolution, Kigali, Rwanda; 5 PATH, Seattle, Washington, USA; 6 Rwanda Biomedical Center, Kigali, Rwanda

**Keywords:** Africa South of the Sahara, Artificial Intelligence, Clinical Decision-Making, Clinical Protocols

## Abstract

**Introduction:**

Community health workers (CHWs) are critical to healthcare delivery in low-resource settings but often lack formal clinical training, limiting their decision-making. Large language models (LLMs) could provide real-time, context-specific support to improve referrals and management plans. This study aims to evaluate the potential utility of LLMs in assisting CHW decision-making in Rwanda.

**Methods and analysis:**

This is a prospective, observational study conducted in Nyabihu and Musanze districts, Rwanda. Audio recordings of CHW-patient consultations will be transcribed and analysed by an LLM to generate referral decisions, differential diagnoses and management plans. These outputs, alongside CHW decisions, will be evaluated against a clinical expert panel’s consensus. The primary outcome is the appropriateness of referral decisions. Secondary outcomes include diagnostic accuracy, management plan quality, and patient and user perceptions to ambient recording of consultations. Sample size is set at 800 consultations (400 per district), powered to detect a 15–20 percentage point improvement in referral appropriateness.

**Ethics and dissemination:**

Ethical approval has been obtained from the Rwandan National Ethics Committee (RNEC) (Ref number: RNEC 853/2025) in June 2025, recruitment started in July 2025 and results are expected in late 2025. Results will be disseminated via stakeholder meetings, academic conferences and peer-reviewed publication.

**Trial registration number:**

PACTR202504601308784.

**STRENGTHS AND LIMITATIONS OF THIS STUDY**

- First prospective evaluation of a large language model-assisted decision support system for community health workers in a real-world, low-resource setting.
- Directly supports Rwanda’s ongoing digital Community Health Worker Application platform rollout.
- The blinded expert panel provides robust reference standards for comparison.
- Study limited to two districts in Rwanda; findings may not be generalisable to other settings.
- Observational design precludes assessing clinical impact on patient outcomes, requiring evaluation in interventional trials.

## Introduction

 Community health workers (CHWs) play a critical role in delivering primary healthcare services in low-resourced and middle-resourced countries, particularly where access to trained medical professionals is often limited.^1^ CHWs act as the first point of contact for communities, providing essential services including triaging, diagnosing and providing basic treatment for a limited number of common health conditions, with a particular focus on maternal, child and infectious disease care.^2 3^

In Rwanda, CHWs typically have limited formal clinical training,^4^ relying on targeted training programmes and following a structured national decision-making algorithm for managing common health conditions.^5 6^ This algorithm has recently been embedded within the newly introduced Community Health Worker Application (CHWApp), a smartphone-based digital decision-support tool, designed to standardise care and guide referrals. The CHWApp rollout forms part of Rwanda’s digital health transformation strategy and, as of December 2024, has been deployed to 1500 CHWs nationwide. However, while digitisation has enhanced record-keeping and streamlined some processes, the clinical decision-making protocols underpinning the CHWApp have remained unchanged. It continues to use a limited, closed-ended decision tree, which may inadequately capture atypical presentations or conditions beyond its predefined scope, potentially negatively affecting patient outcomes and healthcare system efficiency.

Large language models (LLMs) are advanced artificial intelligence (AI) systems trained on extensive text datasets,^3^ enabling them to process and generate human-like text outputs. In healthcare, LLMs have shown potential for improving clinical decision support, diagnostic reasoning and care optimisation.^7 8^ Most published evaluations of LLM performance, however, have taken place in high-income healthcare settings, and little is known about their utility in frontline, low-resource environments, particularly for non-professional healthcare providers like CHWs.^9^

Integrating LLMs into CHW workflows offers the opportunity to provide real-time, context-specific, evidence-based guidance that could improve the appropriateness of referrals and reduce delays in recognising serious conditions. A recent benchmarking exercise evaluating multiple LLMs for alignment with expert medical consensus on questions posed by CHWs demonstrated promising accuracy rates, particularly for newer generation models.^10^ Nonetheless, there remains a critical need for prospective, real-world studies evaluating how LLMs perform when applied to actual patient encounters in low-resource settings.

This study aims to address this evidence gap by prospectively evaluating the appropriateness of referral decisions made by CHWs and an LLM, using a clinical expert panel as the reference standard, in two districts of Rwanda. The findings will inform the feasibility, safety and potential value of integrating LLM-based clinical decision support tools into community healthcare workflows in LMIC settings.

## Methods and analysis

### Study design

This study is a prospective, multisite, observational study designed to evaluate the clinical decision-making performance of CHWs and an LLM in the management of new patient complaints in Rwanda. The study uses real-world patient consultations conducted by CHWs in community settings, with no alteration to existing clinical workflows or patient care pathways. During the study period, CHWs will audio record their routine consultations with eligible patients using a dedicated study application installed on their smartphones. These recordings will be securely uploaded to a central data server and subsequently transcribed. These recordings will be translated from Kinyarwanda to English using an AI-based translation tool. The resulting English transcripts will be anonymised and then provided as input to the LLM, alongside a structured clinical prompt (see online supplemental material 1). The LLM will generate a differential diagnosis list, a referral decision and a proposed management plan for each case. To evaluate the appropriateness of both the CHW decisions (standard of care) and the LLM decisions, a panel of independent general practitioners (GPs), each with a minimum of 2 years of clinical experience in Rwanda, will review each patient consultation transcript using a prespecified, study-specific evaluation rubric.

### Study setting

The study will be conducted in two districts of Rwanda: Nyabihu and Musanze, located in the country’s north-west region. These districts were purposely selected to capture variability in CHW digital tool use and to reflect a range of geographic, socioeconomic and healthcare delivery environments. Nyabihu district represents a largely rural population with over 900 CHWs already using the national smartphone-based CHWApp decision-support platform as part of Rwanda’s digital health transformation strategy. In contrast, Musanze district includes a mix of rural and semiurban communities, and while smartphones have been distributed to 150 CHWs in the area, the CHWApp had not yet been implemented at the time of study commencement. CHWs in Musanze, therefore, continue to use traditional paper-based consultation and referral tools. This diversity in CHW workflow and decision-support mechanisms provides a relevant and representative context to assess the comparative value of LLM decision-support in settings with varying levels of digital health integration.

### Eligibility criteria and recruitment

Eligible CHWs will be identified by UGHE in collaboration with the Rwanda Biomedical Centre (RBC) and district health offices. A Microsoft Excel RAND function will be used to randomly sample 50 CHWs from a list of eligible CHWs in each district. Recruitment will be facilitated via telephone contact by study research assistants (RAs), followed by an in-person consent process conducted at local health centres. The inclusion criteria for CHWs are as follows:

- Completion of RBC national CHW training curriculum.
- At least 3 months of active clinical practice experience.
- Ability to communicate in either Kinyarwanda or English.

The exclusion criteria for CHWs are as follows:

- CHWs out of active service for more than 1 month.
- Intention to relocate or travel outside the study district during the study period.
- Declining or unable to provide informed consent.

CHWs that consent to participate will undergo study-specific training covering the protocol, their role in the study, standard operating procedures, principles of informed consent and trial runs of the study procedures prior to study commencement (see online supplemental material 1).

All patients whose consultations are provided by participating CHWs during the study period will be considered for inclusion. Patient eligibility will be assessed by CHWs, and prior to each consultation, CHWs will provide eligible patients with an explanation of the study and seek written informed consent. CHWs will follow a standardised consent procedure using forms written in Kinyarwanda. Only consenting patients will be enrolled, with no impact on care for those declining participation (see online supplemental material 1). Patient participants attending with the first presentation of any health-related complaint are eligible, including paediatric patients (<18 years of age); those aged 12–18 years will be asked to assent in addition to seeking their guardian’s consent.

Patient participants will be excluded from the study if they are receiving CHW visits for reasons other than new health-related complaints, such as weight checks, vaccinations or routine antenatal care. Patients requiring immediate or emergency medical care, including trauma cases and medical emergencies, will also be excluded. Additionally, non-verbally communicative participants, those in consultation environments that do not allow for audio recording, and patients who do not speak Kinyarwanda or English will not be eligible. Finally, patients who are unable or unwilling to be contacted for follow-up outcomes or to attend a health centre if referred will be excluded from participation.

### Consultation and related events

On consent, the CHW will initiate audio recording of the entire clinical consultation using the StudyApp. CHWs will be instructed to conduct consultations according to their usual practice, guided either by the national CHW decision-making algorithm (on paper or via CHWApp). The consultation recording—and all subsequent LLM outputs—will not be used to influence the care of the patient. The CHW will be asked to provide their usual standard of care, and there are no restrictions placed on that care as a result of this study. Following the consultation, CHWs will administer a brief structured patient experience questionnaire to assess perceptions of being recorded (see online supplemental material 1).

### Follow-up and related events

As part of standard CHW practice, patients will be followed up on day 5 to assess symptom progression and treatment outcomes. In addition, the study protocol includes a day 14 follow-up for research purposes. CHWs will complete a structured follow-up form (see online supplemental material 1) on the StudyApp, capturing information on symptom resolution (resolved, unchanged, worsened, deceased), any further health service use and counter-referral details for referred patients.

Patients reporting full symptom resolution at the day 5 follow-up will not undergo the day 14 follow-up. CHWs will also photograph and upload any counter-referral forms (providing feedback to the CHW for continued care in the community) received from health centres for referred patients (see online supplemental material 1).

After the data collection period, RAs will conduct short user experience surveys and four focus group discussions of 8–12 CHWs to get insight into their experience of being recorded and any challenges faced (see online supplemental material 1).

**Figure 1:**
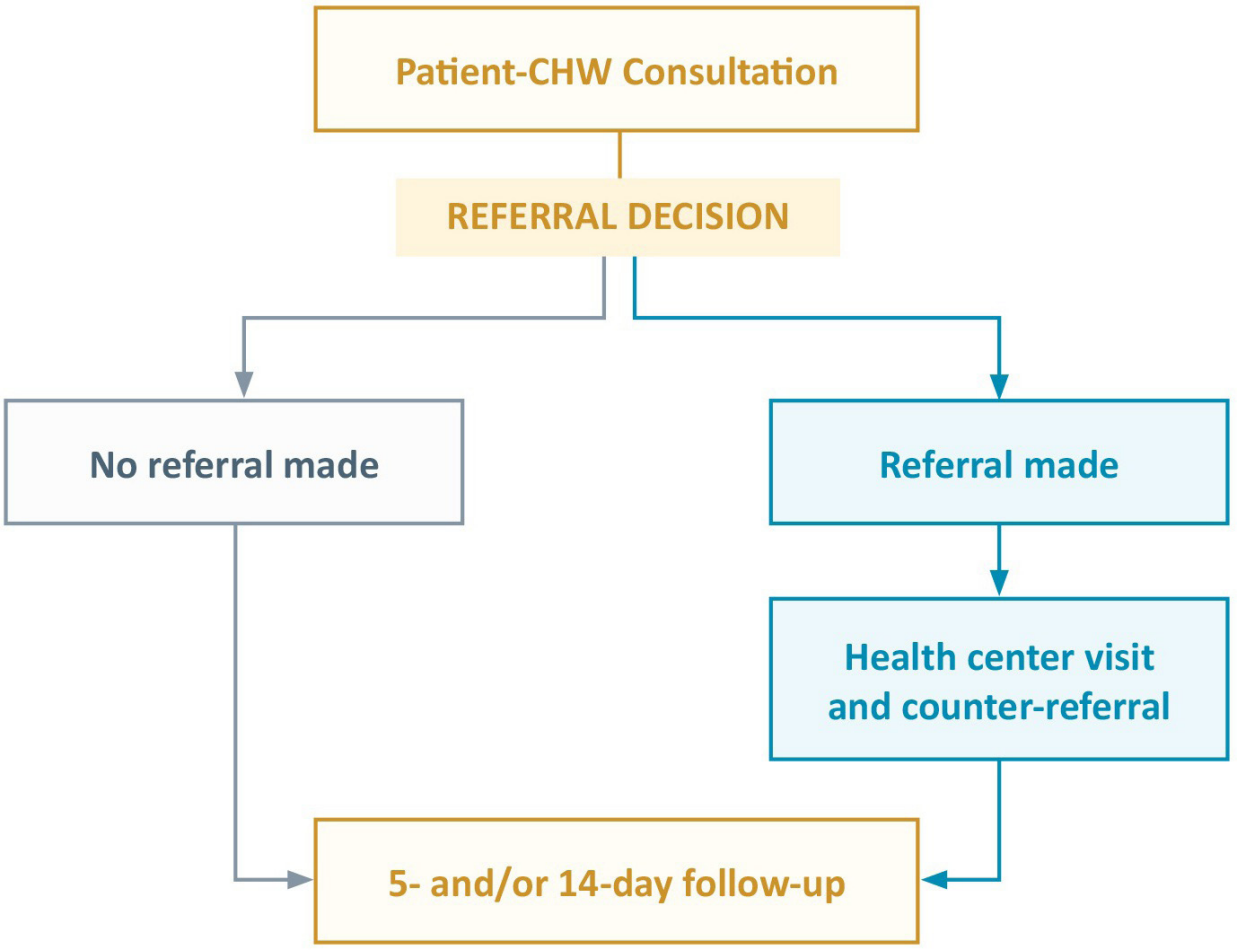
Study participant flow. CHW, community health worker.

Patient participant flow is summarised in figure 1.

### LLM output generation

The recording and transcript will be automatically uploaded and accessed on a secure data warehouse. The first 10–20 transcript samples will be considered a pilot and excluded from the primary analysis. Instead, they will be used to assess recording quality and transcription accuracy, perform exploratory analysis and prompt engineering of the LLM (GPT-4). This process will involve examining the initial transcripts to optimise the LLM’s performance in generating useful outputs and refining how the prompts are structured to elicit the most accurate and relevant responses. By testing and adjusting the prompt structures based on these transcripts, we will effectively ‘tune’ the LLM to improve its clinical decision-making capabilities.

Following this, the research team will omit the interaction’s outcomes (ie, the CHW’s management and referral decision) and feed the transcript and prompts to the LLM to: (1) produce a referral decision, (2) suggest five differential diagnoses in order of likelihood and (3) propose a management plan

### Expert evaluation of CHW outputs and LLM outputs

12 independent GPs, each with a minimum of 2 years of clinical experience in Rwanda and valid Rwanda Medical and Dental Council licences, will be recruited for this study. They will be organised into four panels of three to evaluate each patient–CHW consultation transcript using a study-specific evaluation rubric (see online supplemental material 1). This evaluation will provide a ground truth against which the LLM and CHW outputs will be measured. Evaluators will receive training to standardise the evaluation approach. This training will include an overview of the study protocol and detailed instructions on using the evaluation rubric. During the training, 10 simulated cases will be reviewed to establish a common frame of reference across the panel and to familiarise evaluators with the rubric.

Everyone in the triplet group of evaluators will independently review the case transcript, make a diagnosis and develop a management plan. The evaluator’s tasks for each objective are as follows:

1. Based only on the day 0 consultation transcript, a reference standard should be determined to decide on the appropriateness of the CHW and LLM referral decisions.
2. Review the day 0 consultation transcript and the 5-day or 14-day follow-up information to establish a reference standard for evaluating the correctness of the CHW and LLM referral decisions.
3. Assess the diagnostic accuracy of the LLM through:

-  Establishing one ‘most likely’ diagnosis based on all the information provided.
-  Independently scoring the appropriateness of the differential diagnosis list according to a prespecified rubric.
-  Assessing whether the diagnosis provided in the counter-referral (or, if unavailable, the panel’s ‘most likely’ diagnosis) is the top-ranked or is included within the top five differential diagnoses suggested by the LLM.

4. Assess the appropriateness of the LLM-generated management plan according to the evaluation rubric.

After the initial assessments, the average response for the Likert scale question will be calculated. The panel will also review and discuss the open-ended responses, such as the ‘most likely’ diagnosis, and collaboratively reach a consensus on the diagnosis.

### Primary outcome

The primary outcome of this study is the appropriateness of referral decisions made by CHWs and by the LLM, measured against the consensus decision of an independent expert clinical panel, according to the day 0 consultation transcript only.

For each patient consultation, both the CHW’s actual referral decision on whether to refer the patient to a health facility or manage them in the community and the LLM-generated referral decision will be compared with the reference standard established by a blinded evaluation panel of GPs, based solely on the information available in the day 0 consultation transcript.

### Secondary outcomes

1. Compare the appropriateness of CHW referral decisions vs the LLM referral decisions, using the patient’s 5-day or 14-day outcome and counter referral results as the reference standard.

A secondary assessment of referral decision appropriateness will be performed incorporating patient outcomes at day 5 or day 14 follow-up. For each patient, the expert panel will review both the day 0 consultation transcript and subsequent follow-up outcome data to determine whether the initial referral decision was appropriate in retrospect. The CHW and LLM decisions will again be compared with this revised reference standard.

2. Assess the diagnostic accuracy of the LLM as:

- Appropriateness of the differential diagnosis list—The expert panel will rate the quality of the LLM-generated differential diagnosis list for each case using a predefined 5-point Likert scale rubric, assessing alignment with medical consensus, knowledge recall, omission of important differentials and inclusion of irrelevant differentials (see online supplemental material 1).
- Diagnostic accuracy (top-1 and top-5): For consultations where a definitive diagnosis can be established (either via health centre counter-referral or expert panel consensus), the proportion of cases in which the correct diagnosis appears as the LLM’s top-1 diagnosis or within its top-5 ranked differentials will be reported.

3. Assess the appropriateness of the LLM-generated management plan.

The quality of the LLM’s proposed management plans will be independently evaluated by the expert panel using a 5-point Likert scale predefined rubric, rating appropriateness across five domains including alignment with medical consensus, omission of important information, potential for demographic or contextual bias, likelihood of potential harm, contextual appropriateness relative to available resources for CHWs in Rwanda (see online supplemental material 1).

4. Explore the experience and perspectives of patients and CHWs on being recorded during consultation.

User experience outcomes will assess the acceptability and perceived impact of consultation recording for both patients and CHWs. Immediately after each recorded consultation, patients will complete a brief questionnaire rating their comfort being recorded, perceived effects on communication and consultation thoroughness, and the perceived naturalness of the consultation process (see online supplemental material 1). At the end of the study, CHWs will complete a questionnaire assessing their perceptions of patient comfort, impact of recording on care delivery, ease of using the StudyApp and the need for additional training or technical support. A subset of CHWs will also participate in focus group discussions to explore their experiences in greater depth (see online supplemental material 1).

### Sample size

Sample size estimation for this study was informed by a recent benchmarking study (unpublished at the time of ethics submission) which assessed multiple LLMs on their ability to answer medical questions posed by CHWs. The primary performance metric used in the benchmarking exercise—alignment with expert medical consensus—closely approximates this study’s primary outcome: the appropriateness of referral decisions made by CHWs and the LLM compared with a clinical reference standard.

In the benchmarking dataset, GPs were estimated to make correct referral decisions in 73.3% of cases.^10^ This figure was, therefore, used as a conservative estimate of the referral decision accuracy for CHWs operating without LLM support in our study. In real-world practice, CHW accuracy may be lower than this benchmark, and thus this assumption yields a conservative estimate for sample size requirements by potentially underestimating the true effect size.

The same benchmarking study generated accuracy estimates for five leading LLMs (n=400 cases per model). Accuracy varied across models, with performance reported as follows: Gemini-2 (92.0%), GPT-4o (91.2%), o3 mini (91.0%), Deepseek R1 (83.8%) and Meditron-70B (79.7%). These results suggest that even under conservative assumptions, a performance difference of 15–20 percentage points between CHWs and high-performing LLMs is both plausible and likely detectable.

To estimate the number of interactions required to detect a given improvement in referral decision accuracy, sample size curves were generated for different effect sizes, assuming varying numbers of interactions per CHW per week (based on expected consultation volumes of 7–15 per week). These calculations suggested that, to detect a 15 percentage point increase in referral accuracy over the baseline of 73.3%, between 147 and 1064 total interactions would be required, depending on CHW workload and clustering. To detect a 20 percentage point improvement, between 49 and 392 consultations would be needed. These ranges assumed minimum cluster sizes of 7 interactions per CHW and accounted for clustering in the analysis.

Based on these projections, we plan to collect a total of 800 consultation transcripts, split evenly between two CHW cohorts: 400 recordings from CHWs using paper-based guidelines and 400 from CHWs using the CHWApp digital decision-support tool. To achieve this, we will recruit 50 CHWs per arm, each expected to contribute approximately 10 consultations. This recruitment target allows for a 20% attrition rate, due to technical failure, low recording quality or CHWs submitting fewer than seven recordings (the minimum cluster size for inclusion in analysis). We, therefore, anticipate that 40 CHWs per group will contribute usable data, with an average of 10 transcripts each, yielding the required 400 consultations per arm.

This sample size also allows for robust subgroup analyses and maintains adequate power to detect an absolute difference in referral accuracy as small as 11.8 percentage points—a conservative estimate given the larger effect sizes observed in benchmarking data.

### Statistical analysis plan

Descriptive statistics will be used to summarise the demographic characteristics of participants and key measures.

#### Primary outcome analysis

The primary analysis will involve (1) assessing the LLM’s performance (accuracy) in relation to the ground truth and (2) assessing the LLM’s accuracy compared with the CHW’s performance alone.

**Figure 2:**
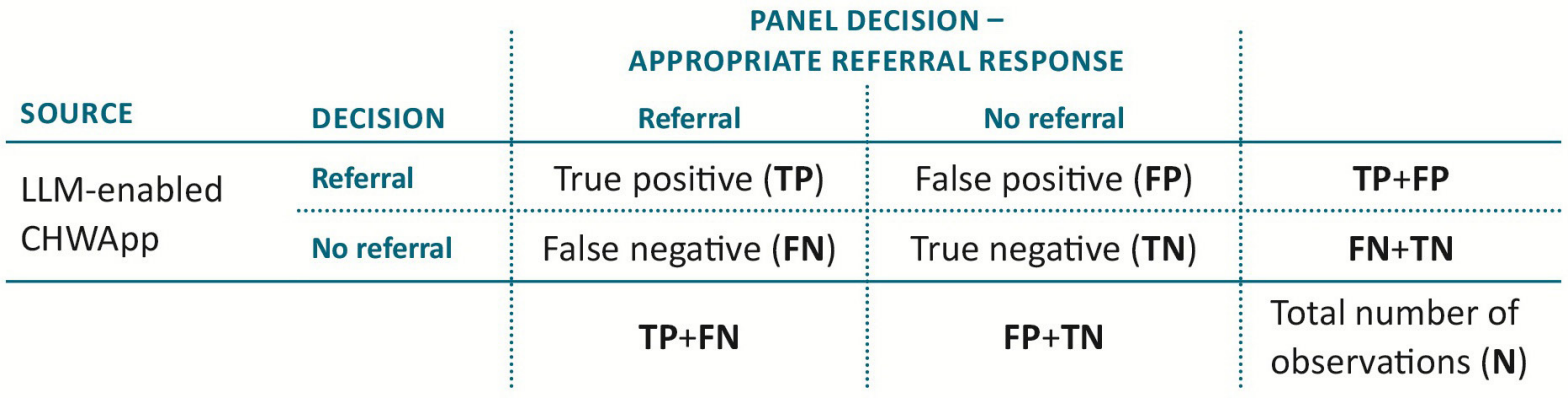
Confusion matrix used in statistical analysis. CHWApp, Community Health Worker Application; LLM, large language model.

All model performance metrics, including accuracy, sensitivity, specificity, positive predictive value and negative predictive value (NPV) and their corresponding confidence intervals will be calculated from information on numbers of true positive, false negative, false positive and true negative model allocations included in the standard 2×2 (confusion) matrix, as shown in figure 2. Note here that the term ‘positive’ indicates a decision to refer the patient, and the term ‘true’ indicates that this decision was appropriate. See online supplemental material for further details on performance metrics.

The performance of the LLM will first be assessed in comparison to the current CHW-only referral approach across the full evaluation data, with no accounting for clustering by geographic location. However, to examine heterogeneity, model performance will also be summarised across natural clusters (defined by CHW as a proxy for geographic location) using a one-stage multivariate meta-analysis approach. Separate one-stage bivariate random-effects logit-normal models will be fitted, with the first jointly synthesising recall and specificity and the second incorporating both precision and NPV.^11 12^ These bivariate meta-analysis models will be extended to incorporate a test-type indicator (LLM vs CHW only) as a meta-regression component to inform the assessment of the difference in referral accuracy between the two referral decision types.^12^ Additional meta-regression components will also be included to adjust the analysis for other important consultation factors, such as whether the consultation involved a paper or app-based decision tree.

A secondary analysis will also be conducted using an extended trivariate model (jointly synthesising recall, specificity and prevalence), with transformations to the predicted probabilities (precision, NPV) following pooling using Bayes Theorem.^12 13^ Values of each of the above performance statistics will be calculated for the LLM and the CHW referral decisions and will be presented with 95% CIs and indications of heterogeneity across CHWs (τ^2^, 95% prediction intervals).

### Secondary outcome analysis

Referral decision appropriateness will be reassessed following integration of day 5 and day 14 follow-up outcome data. The expert panel’s revised appropriateness assessment incorporating these outcomes will serve as an updated reference standard. Diagnostic accuracy metrics and paired comparisons will be repeated against this outcome-informed standard.

For cases with a definitive diagnosis established through expert panel consensus or counter-referral documentation, the proportion of correct diagnoses appearing as the LLM’s top-1 and within its top-5 differential diagnosis list will be reported, with 95% CIs.

Outcomes of interest (appropriateness of differential diagnosis and appropriateness of management plan) will each be measured on a 5-point Likert scale. Distributions of these Likert scale responses will be displayed using frequency tables, numerically summarised across interactions using the median, along with upper and lower quartile values, and visualised using histograms, demonstrating the proportion of interactions rated to be at each level of the scale. For each secondary outcome separately, a multilevel ordinal regression model will be fit, adjusting for important consultation features, with a random effect on the intercept term to account for the natural clustering of the data, as discussed above. Secondary outcome involving measuring the top-1 and top-5 diagnostic accuracy for a subset of participants where a definitive diagnosis was made. The mean (median) rank of the ground truth diagnosis in the differential list will also be summarised.

The patient and CHW user experience questions will be analysed with descriptive analysis to assess the acceptability and impact of recordings. Qualitative responses from CHWs will be thematically analysed to generate themes.

### Data monitoring

All recorded consultations will be automatically uploaded to a secure, password-protected data server hosted by the Centre for the Fourth Industrial Revolution (C4IR) in Kigali. Audio files will be transcribed and translated from Kinyarwanda to English using a purpose-built AI-based translation system developed by a local technology partner.

The LLM’s outputs will be securely stored and linked to the corresponding anonymised consultation record for later comparative analysis.

### Data quality assurance

The quality and completeness of all collected data will be routinely monitored by the study management team. Daily data quality checks will be conducted to verify data upload eligibility, completeness and audibility. Cases with missing history and/or referral outcome in the recording will be automatically discarded. Any missing demographic data will be addressed with the respective CHWs via phone call to fill in retrospectively. Only cases that fulfil the above criteria will be forwarded for transcription, translation and LLM prompting. Follow-up adherence will be assessed weekly. Cases that have no follow-up data will not proceed for evaluation. Instead, CHWs will be prompted to conduct the missing follow-up.

A random sample of 20% of audio recordings will be reviewed by independent Kinyarwanda-speaking researchers to cross-validate machine-generated transcripts, with an acceptable error threshold set at <15%. Any breach of this threshold will trigger a data quality review by the study steering committee to determine whether data collection should continue or changes are required.

## Ethics and dissemination

### Ethical considerations

Ethical approval for this study has been obtained from the Rwanda National Ethics Committee (RNEC) (Approval Number: 853/2025) in June 2025. The study will be conducted in accordance with the principles outlined in the Declaration of Helsinki and adheres to Good Clinical Practice guidelines. Refer to online supplemental material 1 for further details on risk mitigation strategies. If any protocol changes are required in the future, this will be first submitted for approval to RNEC and the Sponsor, prior to notification of all relevant study staff and updating the listed protocol on the PATH website.

All participating CHWs and patients will provide written informed consent prior to study enrolment. CHWs will be consented in person by trained RAs during the enrolment process. Patients will be informed about the purpose of the study, the voluntary nature of participation, their right to withdraw at any point without any impact on their care, and the use of audio recordings for research purposes. Consent materials will be provided in both Kinyarwanda and English.

For minors (age <18 years), consent will be obtained from a parent or legal guardian, with assent sought from children aged between 12 and 18 years. CHWs will be trained in ethical consent procedures to ensure patient understanding and voluntariness.

### Data privacy and confidentiality

All collected data, including audio recordings, transcripts, LLM outputs and evaluation panel assessments, will be deidentified at the point of collection. Unique study identifiers will be assigned to each consultation record to prevent the use of personally identifiable information. Audio files will be encrypted and stored on a secure, password-protected server hosted by the C4IR in Kigali, Rwanda. Only authorised study personnel will have access to the data.

Data will not be shared with third-party AI model developers or used for any purposes beyond the scope of this study. All LLM analyses will be conducted within a secure, controlled environment to minimise data security risks.

## Discussion

The evidence base for the use of LLMs in frontline healthcare delivery remains limited, particularly in low-resource countries where the potential benefits of AI-driven decision support may be most impactful. This lack of context-specific evidence presents a challenge for policy-makers and implementers seeking to understand which AI tools are safe, effective and appropriate for integration into existing community health systems.

To address this gap, we have designed and launched a pragmatic, prospective observational study evaluating the potential utility of LLM-based clinical decision support for CHWs in Rwanda. By directly comparing the LLM outputs against both a gold standard (expert clinical consensus), and against the current standard of care (CHW decision alone), this study aims to generate critical evidence on the feasibility, safety and contextual appropriateness of LLM-assisted care at the community level.

While the study has several inherent limitations—such as its observational design and limited geographical footprint—we will transparently report these limitations and discuss their implications for the interpretation and generalisability of findings. This protocol outlines key methodological choices made to balance scientific rigour with feasibility in a low-resource environment and aims to contribute to the growing discourse on evaluating AI interventions in primary care settings in Africa.

Data collection commenced in July 2025, and we anticipate that final study results will be available for dissemination by December 2025.

## Supplementary material

10.1136/bmjopen-2025-110927 online supplemental file 1
