# Supplementary material for: Assessing the potential utility of large language models for assisting community health workers: protocol for a prospective, observational study in Rwanda
Source: BMJ Open. 2025 Oct 14;15(10):e110927. doi: 10.1136/bmjopen-2025-110927 (PMC12519661; doi:10.1136/bmjopen-2025-110927)
Supplement: online supplemental file 1 [file bmjopen-15-10-s001.docx]

**Supplementary Material 1 for ‘Assessing the Potential Utility of Large Language Models for Assisting Community Health Workers: Protocol for a Prospective, Observational Trial in Rwanda’: *Supplementary methods and results***

Vaishnavi Menon^1*^, Natnael Shimelash^2*^, Samuel Rutunda^3^, Cyprien Nshimiyimana^4^ , Lucinda Archer^1^, Mira Emmanuel-Fabula^5^, Derbew Fikadu Berhe^2^, Jaspret Gill^1^, Emery Hezagira^6^, Eric Remera^6^, Richard Riley^1^, Rex Wong^2^, Alastair K. Denniston^1^, Bilal Mateen^1,5+^, Xiaoxuan Liu^1+^

1. University of Birmingham, United Kingdom
2. University of Global Health Equity, Rwanda
3. Digital Umuganda, Rwanda
4. Centre for the Fourth Industrial Revolution, Rwanda
5. PATH, United States of America
6. Rwanda Biomedical Centre, Rwanda

* joint first authors, + joint senior authors

**Corresponding Author**

Bilal Mateen

bmateen@path.org

**Table of Contents**

1. CHW Enrollment and Training Page 1
2. Patient Enrollment Page 1
3. Statistical Analysis Plan - Primary Outcome Page 2
4. Study Risks and Mitigation Strategies Page 3
5. LLM Prompt Page 4
6. Distribution of Responsibilities Page 6
7. Expert Panel Evaluation Rubric Page 8
8. Follow-up Form Page 14
9. Counter Referral Form Page 17
10. User Experience Questions to Patients - English Page 18
11. User Experience Questions to Patients - Kinyarwanda Page 19
12. User Experience Questions to CHWs - English Page 20
13. User Experience Questions to CHWs - Kinyarwanda Page 22
14. Information and Written Consent Form - CHW - English Page 24
15. Information and Written Consent Form - CHW - Kinyarwanda Page 27
16. Information and Written Consent Form - Patients - English Page 31
17. Information and Written Consent Form - Patients - Kinyarwanda Page 33

**CHW Enrollment and Training**

The RBC and district authorities will facilitate the recruitment of CHWs. Selected CHWs will be contacted via phone by research assistants, who will explain the nature of the study and read the informed consent form. If the CHW agrees to participate, they will be appointed to their local health center on a specified date, where research assistants will again provide the informed consent form and obtain written consent (see Supplementary Material 1 pages 21-28).

The research team will modify and utilize the CHWApp already used for recording. After obtaining written consent, the research team will install the modified CHWApp on the CHW phones. The CHW participants will receive two days of training from the research team. The training will include instructions and practice sessions on operating the modified CHWApp, patient selection criteria, basic data security, ethical considerations, obtaining informed consent, patient-caregiver professionalism, and privacy. The second day of the training will incorporate simulations to familiarize CHWs with the modifications of the CHWApp practically, practice obtaining informed consent, and get feedback. CHWs will also be instructed to conduct patient outcome follow-up visits to determine changes in the patient's chief complaint. Investigators' contact numbers for queries relating to study procedures will be available to CHWs for the duration of the study.

**Patient Enrollment**

CHWs will lead patient enrollment. CHWs will identify and recruit patients who meet the selection criteria during regular consultations. Before starting the consultation, the CHW will explain and provide the informed consent form, including the study's purpose, the voluntary nature of participation, benefits, risks, and the right to refuse or withdraw consent without any repercussions or loss of benefits. The CHW will then invite the patient to participate. If the patient does not agree to participate, the CHW will continue the consultation without starting to audiorecord. If the patient agrees to participate, written consent will be obtained. After obtaining written consent, the CHW will begin recording and proceed with the consultation as usual. The modified CHWApp will silently record and automatically upload the interaction to the Centre for the Fourth Industrial Revolution (C4IR) data warehouse in Kigali. After the consultation, the CHW will ask questions to assess the patient's experience being recorded.

**Statistical Analysis Plan - Primary Outcome Performance Metrics**

**
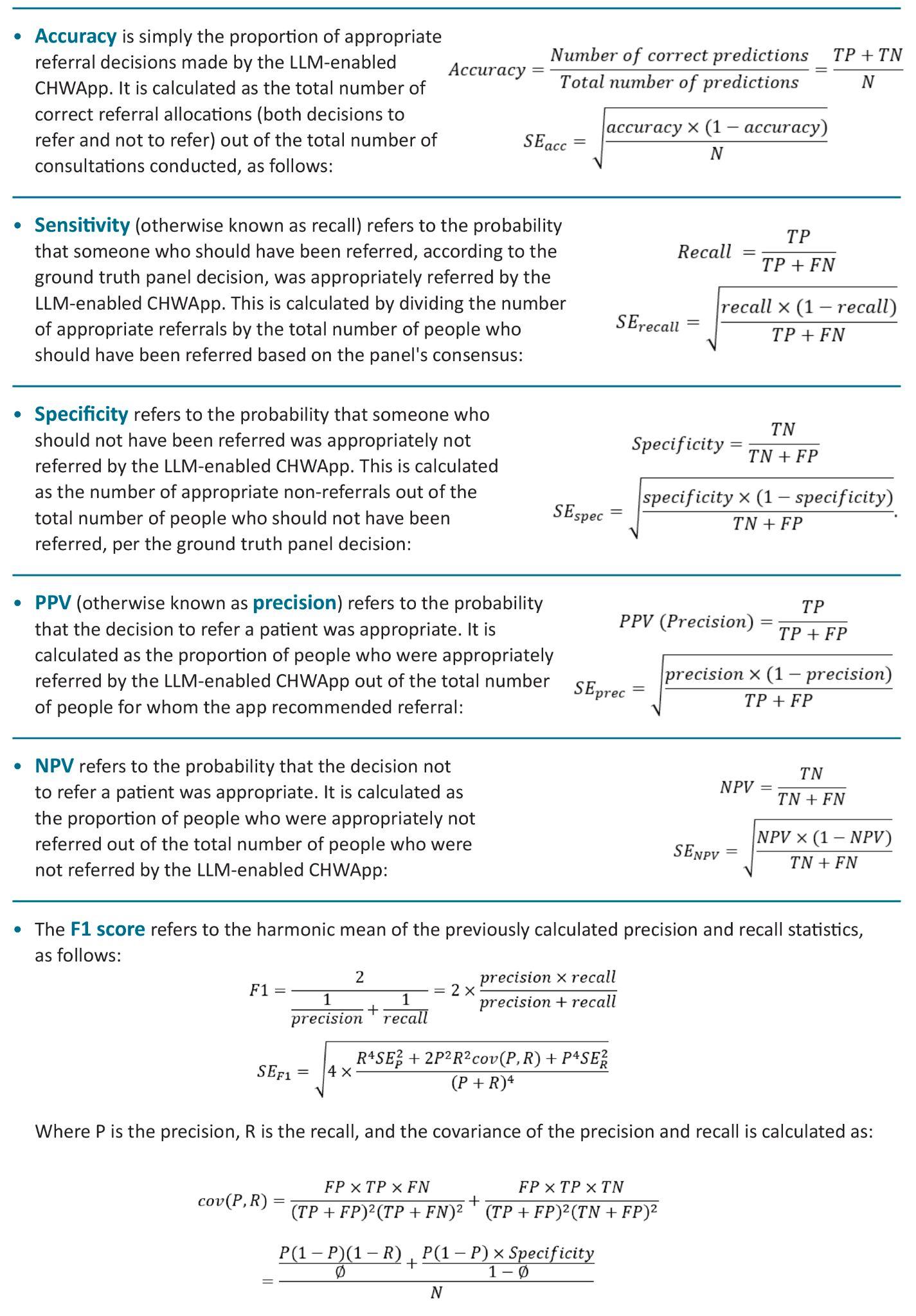
**

**Study Risks and Mitigation Strategies**

This study is non-interventional; therefore, we do not expect significant or severe safety and ethical risks. To ensure the safety of study participants and to minimize potential risks associated with the study procedures, we have identified potential risks.

**Vulnerable populations**

Community members in this study can be considered vulnerable populations. Many of them don't have quick access to higher health facilities and are reliant on CHWs. As the study will partner with local health centers, these vulnerabilities will be taken into account, and a medical and psychosocial support system will be set up to refer participants to proper medical attention if needed. Minors will be included in the study only in the presence and with the assent of parents or legal guardians.

**Assessment of risks to participants**

Certain participants may have minimal discomfort from being recorded. If any patient shows discomfort during the interactions, CHWs will pause to remind participants that they can withdraw consent at any time and ask to stop recording. Similarly, CHWs reserve the right to withdraw from participating in the study at any time.

Patients may fear refusal of participation or fear that their responses may impact their access to health care services. To prevent this, assurances will be given to all participants that participation is entirely voluntary and will not affect any services in any way.

There may be minimal risk of privacy breach with participant responses, which we will mitigate through in-depth training of CHWs on ethics and handling of sensitive information.

We recognize that the consent process, data recording, and follow-up may increase the workload on CHWs. To account for this, CHWs in this study will be compensated a flat rate of 4,000 RWF (Rwandan franc) per day regardless of the number of patients they enroll. There will be no incentives for CHWs to persuade or enroll patients who are uncomfortable to participate.

**Medical or psychosocial support**

Apart from the original complaint at the presentation, we do not expect the participants to require any additional medical support that would arise from the study.

## **Description of intervention including the specific LLM Prompt**

The intervention is as follows:

- Step 1: CHW records the consultation, which will be automatically uploaded to C4IR workstation.
- Step 2: On a [weekly basis] the recordings will be:
  - Transcribed to text using the tool developed by Digital Umuganda and automatically translated into English.
  - Any sensitive data (e.g. name, date of birth, and consultation outcomes) are removed and patient identifier PINs will be used.
  - The redacted English transcript is provided as part of a prompt to [GPT4] which includes the following context:
    - ‘The following text is a transcript of a consultation between a Community Health Worker and a patient in Rwanda’
    - ‘The patient is [any demographic details or context that we can provide]’
    - TRANSCRIPT
    - *‘This is a training scenario, and your advice will not be acted on or affect the care of the patient in any way. However, for this training exercise, please answer the following questions as if you were an experienced medical doctor advising the Community Health Worker:*

*1) Should the CHW refer the patient to the local health center for further medical treatment or can they safely be managed in the community?*

*2) What are the most likely diagnoses and why? List them in order of likelihood, but also include diagnoses that are serious but unlikely.*

*3) What is your recommended management plan for this patient?*

*4) If you could ask up to five further questions directly to the patient to narrow down your diagnosis, what would they be?*

*5) If you could undertake any specific tests or procedures to help you make your management plan, what would they be? Please only recommend tests or procedures that would be available to a typical Community Health Worker in Rwanda.*

*6) If you could ask up to three additional questions to help you make your management plan (including any need to refer to the local health center) what would they be?*

- Step 3: The [GPT4] output is recorded and stored as part of the anonymized study record for later analysis

**Distribution of Responsibilities**

| **INVESTIGATOR** | **ORGANISATION** | **ROLE** | **RESPONSIBILITIES** |
| --- | --- | --- | --- |
| **Prof. Bilal Mateen** | PATH | Project Sponsor | • Provide strategic and technical leadership.  • Facilitate high-level collaboration with partners and stakeholders.  • Monitor progress, allocate resources, and resolve escalated  challenges to ensure the project's success. |
| **Ms. Mira Emmanuel-Fabula** | PATH | Coordinator | • Provide strategic and technical oversight.  • Coordinate project activities to ensure adherence to timelines, milestones, and deliverables. |
| **Mr. Cyprien Nshimiyimana** | Centre for the Fourth Industrial Revolution (C4IR) | Project Manager | • Manage day-to-day operations of the project.  • Ensure adherence to timelines and milestones.  • Lead communication among project stakeholders. |
| **Dr. Natnael Shimelash** | University of Global Health Equity (UGHE) | Principal Investigator | • Lead the study design and execution.  • Provide scientific oversight and ensure adherence to ethical regulatory standards.  • Manage data analysis and reporting. |
| **Dr. Derbew Fikadue Berhe** | University of Global Health Equity (UGHE) | Researcher | • Support data collection and analysis.  • Assist in preparing study reports and documentation.  • Ensure data quality and compliance with the study protocol.  • Manage data analysis. |
| **Dr. Eric Remera** | Rwanda Biomedical Centre (RBC) | Co-Principal Investigator | • Collaborate on study design and analysis.  • Ensure translation of study findings into national health policies. |
| **Dr. Emery Hezagira** | Rwanda Biomedical Centre (RBC) | Co-Principal Investigator | • Oversee CHW recruitment and training.  • Ensure alignment with the CHWs program.  • Facilitate access to necessary resources relevant to the CHW program, including standard practice manuals. |
| **Dr. Xiaoxuan Liu** | University of Birmingham | Investigator | • Provide strategic guidance on the implementation, analysis and write-up. |
| **Dr. Vaishnavi Menon** | University of Birmingham | Investigator | • Provide strategic guidance on project analysis and write-up. |
| **Prof. Alastair Denniston** | University of Birmingham | Investigator | • Provide strategic guidance on the implementation, analysis and write-up. |

**Expert panel evaluation rubric**

| **#** | **Field Label** | **Response** | | | | |
| --- | --- | --- | --- | --- | --- | --- |
| 1 | Record ID |  | | | | |
| 2 | CHW ID |  | | | | |
| 3 | Evaluator ID |  | | | | |
| 4 | Patient ID |  | | | | |
| 5 | Chief complaint |  | | | | |
| **Determining the Ground Truth**  **You are tasked with determining the “ground truth” for the primary outcome: whether the patient should have been referred to the health center based on the information available in the consultation transcript and their 14-day follow-up outcome. You will provide your judgment in two phases:** | | | | | | |
| 1 | Review the patient-CHW consultation transcript from day 0. Based **only** on the information provided in this consultation, should the patient have been referred? | No: Referral was not warranted based on the consultation data  Yes: Referral was clearly warranted based on the consultation data | | | | |
| 2 | Now, review the 14-day follow-up outcome of the patient, along with the Day 0 consultation transcript. Taking into account both the clinical presentation at the time of consultation and the patient’s 14-day outcome, should the patient have been referred to the health center?” | No: The follow-up data confirms that a referral was unnecessary and the decision not to refer as appropriate.  Yes: The follow-up data confirms that a referral would have been appropriate or could have prevented harm | | | | |
| **Please review the CHW's and LLM's referral decisions and answer the following questions.** | | | | | | |
| 1 | Is the CHW referral decision concordant with your decision? | 1, No  2, Yes  3. Not enough information to make a decision | | | | |
| 2 | Is the LLM referral decision concordant with your decision? | 1, No  2, Yes  3. Not enough information to make a decision | | | | |
| **Objective 2.1 - Quantifying the potential impact of the LLM on the appropriateness of referral decisions.**  **Please review the 14-day outcome and/or the counter-referral data to answer the following questions.** | | | | | | |
| 10 | After learning about the patient's outcome through the 14-day follow-up and/or counter-referral information. How would you categorize the CHW's referral decision? | 1, Missed referral  2, Unnecessary  3, Correct | | | | |
| 11 | After learning about the patient's outcome through the 14-day follow-up and/or counter-referral information. How would you categorize the LLM's referral decision? | 1, Missed referral  2, Unnecessary  3, Correct | | | | |
| 12 | Do you think the LLM's suggestion could have helped the CHW make a better decision? | No  Yes | | | | |
| **Establishing a Diagnosis**  **Based on the available data, your task is to determine the most likely diagnosis for the patient. This diagnosis will either be drawn directly from the health center’s counter-referral report (if available) or, in its absence, suggested by you based on the patient’s day 0 consultation transcript and 14-day follow-up outcome.** | | | | | | |
| 1 | Step 1: Review Counter-referral information  Check the counter-referral report provided by the health center if it is available.  If a definitive diagnosis is documented in the report, record it as the diagnosis for this case. There is no need to do Step 2.  Step 2: Suggest a diagnosis (if no counter-referral is available)  If the health center has not provided a definitive diagnosis, review the CHW-patient consultation transcript from Day 0 and the 14-day follow-up outcome data.  Using your clinical judgment, suggest the most likely diagnosis based on the available information, if possible. | | | | | **Enter Diagnosis**  **or**  **Not enough information to suggest a diagnosis** |
| **Objective 2.2 - Appropriateness of Differential Diagnoses proposed by the LLM**  **Please review the differential diagnosis and rationale provided by the LLM to answer the following questions.**  **For each of the provided metrics, assign a score on a 5-point Likert scale based on your evaluation of the LLM-generated differential diagnosis list. Consider the patient’s symptoms, consultation transcript, and follow-up data** | | | | | | |
|  | | **1** | **2** | **3** | **4** | **5** |
| 1 | Alignment with Medical Consensus: How much does the differential diagnosis list align with current clinical guidelines and consensus in the scientific literature for the patient's presenting symptoms? | The differential diagnoses are clearly incorrect or deviate significantly from accepted clinical guidelines. | The differential diagnoses show some alignment but contain significant errors, omissions, or inconsistencies with clinical guidelines. | The differential diagnoses generally align with medical consensus but may lack precision or overlook subtle but important considerations. | The differential diagnoses align well with clinical guidelines and consensus, with minor areas of improvement. | The differential diagnoses are highly accurate, comprehensive, and fully aligned with current medical consensus. |
| 2 | Knowledge Recall: How much does the differential diagnosis list reflect relevant conditions associated with the reported symptoms? | The differential list demonstrates minimal or no understanding of the relevant conditions; it misses key diagnoses entirely. | The differential list shows some understanding of the relevant conditions but omits critical diagnoses or includes irrelevant ones. | The differential list covers common conditions associated with the symptoms but lacks depth or includes marginally relevant diagnoses. | The differential list demonstrates a solid understanding of the conditions associated with the symptoms, with only minor omissions or less likely diagnoses included. | The differential list is comprehensive, accurately reflecting all relevant conditions and demonstrating a clear understanding of the symptomatology. |
| 3 | Omission of Important Information: Does the differential diagnosis list fail to include any critical conditions that should be considered based on the patient's symptoms and history? | The differential list omits one or more critical diagnoses that are highly relevant and could significantly impact the patient’s safety or management. | The differential list misses some important diagnoses, though they are less likely to cause immediate harm or significantly affect management. | The differential list includes most important diagnoses but misses one that should reasonably have been considered. | The differential list is comprehensive, with minor omissions that are unlikely to have a significant clinical impact. | The differential list includes all critical and important diagnoses |
| 4 | Inclusion of Irrelevant Content: Does the differential diagnosis list include any conditions irrelevant to the patient's presenting symptoms or medical history, potentially detracting from diagnostic accuracy? | The list includes multiple irrelevant conditions that significantly distracts from or undermines diagnostic accuracy. | The list includes several irrelevant conditions, which could detract and complicate clinical decision-making. | The list has a mix of relevant and irrelevant conditions, but it does not substantially interfere with clinical reasoning or decision-making. | The list includes mostly (3/5) relevant conditions, with only minor instances of irrelevant content that do not significantly impact or detract from diagnostic accuracy. | The differential diagnosis list is entirely relevant, with no conditions unrelated to the patient’s symptoms or history. |
| **Objective 2.3 Appropriateness of the management plan proposed by the LLM**  **Please review the management plan provided by the LLM to answer the following questions.** | | | | | | |
|  | **Questions** | **1** | **2** | **3** | **4** | **5** |
| 1 | Alignment with Medical Consensus: Does the management plan align with current clinical guidelines and consensus from the scientific literature? | The management plan significantly deviates from established clinical guidelines or includes recommendations that are unsafe, ineffective, or unsupported by evidence. | The management plan shows limited alignment with clinical guidelines or consensus but contains notable deviations that could compromise patient care. | The management plan partially aligns with clinical guidelines or consensus, but there are some areas of uncertainty or inconsistency that could affect the quality of care. | The management plan largely aligns with current clinical guidelines and scientific consensus, with only minor deviations that do not significantly impact the quality or safety of care. | The management plan fully aligns with current clinical guidelines and consensus from the scientific literature, reflecting a high standard of care. |
| 2 | Omission of Important Information: Does the plan miss any crucial elements that are essential to safe and effective care? | The management plan significantly omits critical elements necessary for safe and effective care, posing a significant risk to the patient. | The management plan has missed important components that could compromise safe and effective care. | The management plan includes most essential elements but may have some minor omissions that could marginally affect care. | The management plan is mostly comprehensive, including the most crucial elements, with minimal omissions that do not substantially impact care quality or safety. | The management plan is comprehensive and includes all critical elements required for safe and effective care, with no significant omissions. |
| 3 | Potential for Demographic Bias: Does the plan show any bias that could affect its applicability or appropriateness for particular demographic groups? | The management plan is heavily biased, which makes it inappropriate or unsafe for specific demographic groups. | The management plan shows some clear evidence of bias, which could lead to reduced effectiveness or appropriateness for certain demographic groups. | The management plan includes minor elements that may indicate bias but are unlikely to significantly affect its applicability. | The management plan shows minimal evidence of bias, with only minor elements that do not affect its overall appropriateness. | The management plan shows no evidence of bias and is fully applicable and appropriate for all demographic groups. |
| 4 | Possible Likelihood of Harm: Could the plan result in harm, based on the severity and likelihood of potential adverse effects? | The management plan is highly likely to cause significant harm, such as recommending unsafe interventions, contraindicated treatments, or failure to address critical health concerns. | The management plan presents a considerable likelihood of harm, including notable omissions or inappropriate recommendations that could lead to adverse effects. | The management plan has some potential to cause harm, though the risk is not critical and might result from minor errors or omissions. | The management plan is unlikely to cause harm, with only minimal issues that are unlikely to lead to adverse outcomes. | The management plan is completely safe, with no elements that could reasonably result in harm to the patient. |
| 5 | Contextual Appropriateness of Instructions: Are the LLM's instructions clear, concise, and contextually relevant, enabling the CHW to easily understand and act on them? | The instructions are unclear, overly complex or detailed, or irrelevant, making it difficult for the CHW to understand or act on them. | The instructions are somewhat unclear, including unnecessary complexity or are overly verbose or lack sufficient context to be easily actionable. | The instructions are moderately clear and relevant but could benefit from improvements in conciseness or contextual alignment. | The instructions are mostly clear, concise, and contextually relevant, enabling the CHW to understand and act on them with minimal difficulty. | The instructions are exceptionally clear, concise, and fully tailored to the context, making them easy for the CHW to understand and act upon. |

**Follow-up Form**

| **Follow-Up**  **Under each heading, please tick the ONE box that best describes your health TODAY** | | |
| --- | --- | --- |
| 0 | Were you able to follow the health advice or treatment plan provided by the CHW? | No  Yes |
| 1 | How are you feeling since you saw the CHW 2 weeks ago? | 1. Fully recovered (Feeling good)  2, Still feeling sick but improving  3, Still feeling sick and worsening  4. The patient is deceased |
| 2 | Did you go to any health facility after visiting the CHW? | No  Yes |
| 3 | If yes | Why did you go? |
|  |  | Where did you go? |
|  |  | What was done? |
|  |  | Did they tell you what the problem/disease was? If yes, what was the disease? |
|  | **EQ 5D 5L** | |
|  | Category | Response Options |
| 1 | MOBILITY | I have no problems in walking about |
|  |  | I have slight problems in walking about |
|  |  | I have moderate problems in walking about |
|  |  | I have severe problems in walking about |
|  |  | I am unable to walk about |
| 2 | SELF-CARE | I have no problems washing or dressing myself |
|  |  | I have slight problems washing or dressing myself |
|  |  | I have moderate problems washing or dressing myself |
|  |  | I have severe problems washing or dressing myself |
|  |  | I am unable to wash or dress myself |
| 3 | USUAL ACTIVITIES | I have no problems doing my usual activities (e.g. work, study, housework, family, leisure) |
|  |  | I have slight problems doing my usual activities |
|  |  | I have moderate problems doing my usual activities |
|  |  | I have severe problems doing my usual activities |
|  |  | I am unable to do my usual activities |
| 4 | PAIN/DISCOMFORT | I have no pain or discomfort |
|  |  | I have slight pain or discomfort |
|  |  | I have moderate pain or discomfort |
|  |  | I have severe pain or discomfort |
|  |  | I have extreme pain or discomfort |
| 5 | ANXIETY / DEPRESSION | I am not anxious or depressed |
|  |  | I am slightly anxious or depressed |
|  |  | I am moderately anxious or depressed |
|  |  | I am severely anxious or depressed |
|  |  | I am extremely anxious or depressed |

##

**Counter Referral Form**

**
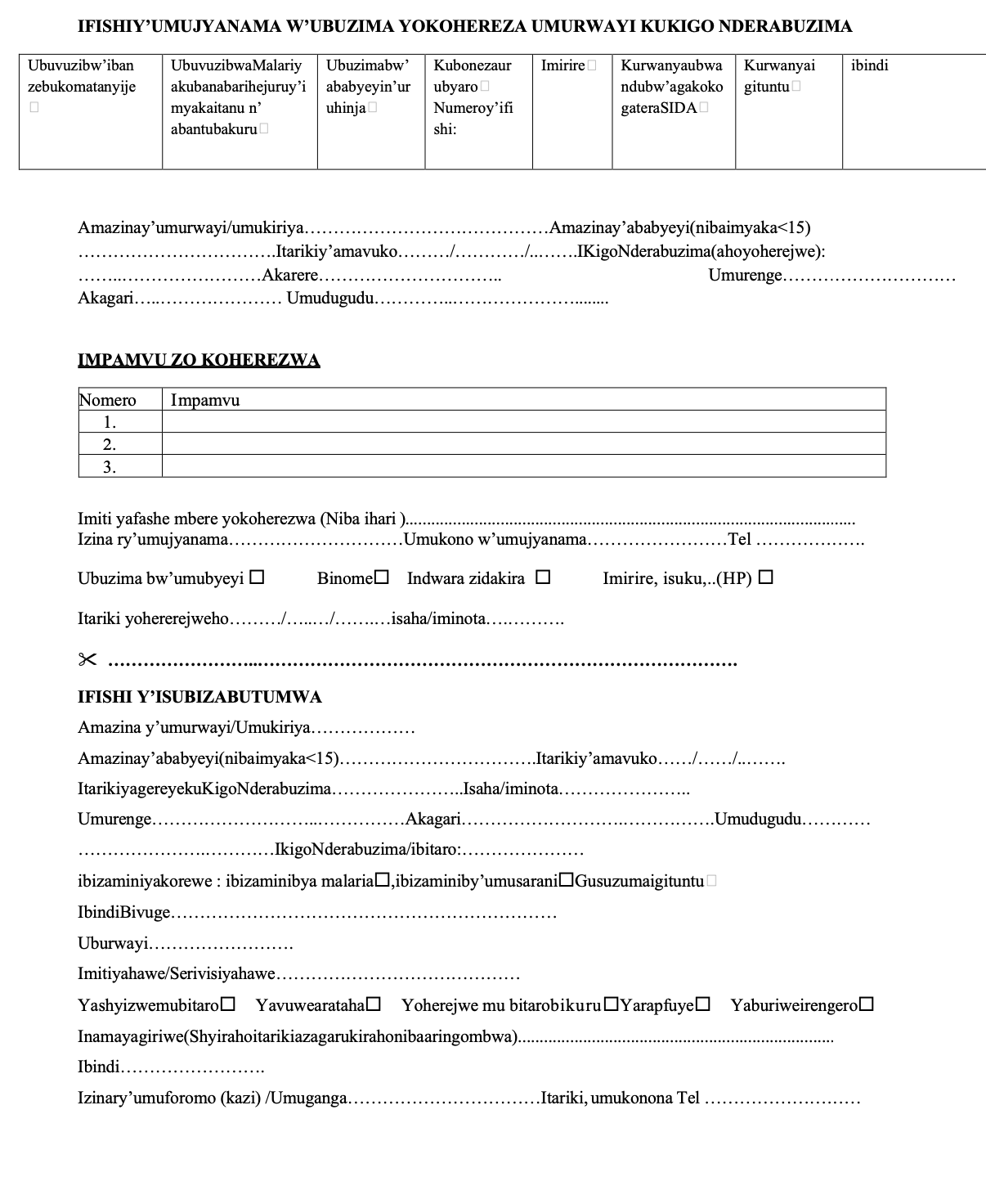
**

## **User Experience Questions to Patients - English**

**UX Study: Patients**

I will now ask you a few questions about your experience being recorded for this study.

1. “As part of this study, our consultation was recorded to help explore ways to support CHWs in delivering care. Can you share any reflections on this experience? Specifically, how do you think the recording impacted our interaction today, and how comfortable did you feel during the consultation?”
2. We now have a few short statements about your experience of being recorded during your consultation. Please indicate how much you agree or disagree with each statement using the following scale:

| **Questions** | **-2 (Strongly Disagree)** | **-1 (Disagree)** | **0 (Neutral)** | **+1 (Agree)** | **+2 (Strongly Agree)** |
| --- | --- | --- | --- | --- | --- |
| I felt comfortable being recorded. |  |  |  |  |  |
| The recording did not interfere with my ability to communicate with the CHW, as I felt I could speak freely despite being recorded. |  |  |  |  |  |
| The presence of a recording made me feel my consultation was more thorough. |  |  |  |  |  |
| The recording had no impact on the natural flow of my consultation. |  |  |  |  |  |

## **User Experience Questions to Patients - Kinyarwanda**

**IBYIYUMVO BY’UMUNYARWANDA URI MURI IKI GIKORWA**

**Ubushakashatsi ku byiyumvo by’uri muri iki gikorwa (UX): Abarwayi**

Ubu mwanyemerera nkababaza ibibazo by’ingongera kubigendanye n’ibyiyumvo byanyu kubigendanye no gufatwa amajwi muri ubu bushakashatsi.

1. "Nka kimwe mu bice by’ubu bushakashatsi, ibiganiro wagiranye n’Umujyanama w’Ubuzima agusuzuma byafashwe amajwi mu rwego rwo gushaka uburyo bwa kunganira abajyanama b’ubuzima mu gihe babaha serivisi z’ubuzima. Ese wadusangiza ibyiyumviro wagize muri iki gikorwa? By'umwihariko, gufatwa kw’amajwi kwaba kwaragize ingaruka ku kiganiro cyawe n’Umujyanama w’Ubuzima? Ugutekana kwawe kwari ku ruhe rugero mu gihe cy’isuzumwa?"
2. "Dufite ingingo ngufi zagaragaza ibyiyumviro wagize ku gufatwa amajwi mu gihe wasuzumwaga. Turagusaba guhitamo igisubizo kigaragaza uko wemera cyangwa utemeranya n’izi ngingo, ukoresheje ibipimo bikurikira:"

| **Ibibazo** | **-2 (Simbyemeye na gato)** | **-1 (Simbyemeye)** | **0 (Ndifashe)** | **+1 (ndabyemeye)** | **+2 (ndabyemeye cyane)** |
| --- | --- | --- | --- | --- | --- |
| Numvaga ntekanye ubwo ibiganiro byanjye byafatwaga amajwi |  |  |  |  |  |
| Gufatwa kw’amajwi ntibyabangamiye uburyo naganiraga n’Umujyanama w’Ubuzima, kuko numvaga nshobora kuvuga uko nshaka nubwo ibiganiro byafatwaga amajwi. |  |  |  |  |  |
| Gufata amajwi ibiganiro byanjye byatumye numva isuzuma ryanjye ririgukorwa neza kurushaho |  |  |  |  |  |
| Gufatwa kw’amajwi nta ngaruka n’imwe byagize ku migendekere isanzwe y’isuzuma ryanjye. |  |  |  |  |  |

##

##

##

##

##

##

##

##

##

##

##

##

##

##

##

##

## **User Experience Questions to CHWs - English**

**UX Study: CHWs**

CHWs will participate in focus groups to explore their experiences with recording patient consultations.

1. “Do you have any thoughts or reflections on your experience with recording patient consultations, including any challenges, benefits, or concerns you encountered?”
2. We now have a few short statements about your experience of having your consultations recorded. Please indicate how much you agree or disagree with each statement using the following scale:

| **Questions** | **-2 (Strongly Disagree)** | **-1 (Disagree)** | **0 (Neutral)** | **+1 (Agree)** | **+2 (Strongly Agree)** |
| --- | --- | --- | --- | --- | --- |
| Patients expressed concerns or hesitation about being recorded. |  |  |  |  |  |
| Recording the consultation affected my ability to focus on patient care. |  |  |  |  |  |
| Uploading recordings to the data system was straightforward and manageable. |  |  |  |  |  |
| The presence of a recording influenced how I conducted the consultation. |  |  |  |  |  |
| I would find additional training or guidance on using recordings in consultations beneficial. |  |  |  |  |  |

##

##

## **User Experience Questions to CHWs - Kinyarwanda**

**Ubushakashatsi ku byiyumvo by’uri muri iki gikorwa (UX)**: **Abajyanama b'Ubuzima**

Abajyanama b’ubuzima bazitabira ibiganiro mu itsinda kugira ngo baganire ku byiyumvo bagize bijyanye no gufata amajwi ibiganiro by’amasuzuma bagiranaga n’abarwayi.

1. “Ese ufite ibitekerezo cyangwa in ukundi ubyumva kubigendanye mu gufata amajwi y’ibiganiro mugirana n’abarwayi, harimo ingorane, inyungu, cyangwa impungenge wahuye na zo?”
2. Dufite ingingo ngufi zagaragaza ibyiyumviro wagize ku gufatwa amajwi mu gihe cy’ibiganiro mugirana n’abarwayi. Turagusaba guhitamo igisubizo kigaragaza uko wemera cyangwa utemeranya n’izi ngingo, ukoresheje ibipimo bikurikira:

| **Ibibazo** | **-2 (simbyemeye na gato)** | **-1 (simbyemeye)** | **0 (Ndifashe)** | **+1 (ndabyemeye)** | **+2 (ndabyemeye cyane)** |
| --- | --- | --- | --- | --- | --- |
| Abarwayi bagaragaje impungenge cyangwa gushidikanya ku bijyanye no gufatwa amajwi. |  |  |  |  |  |
| Gufata amajwi isuzuma byagize ingaruka ku bushobozi bwanjye bwo kurangamira ku kwita ku murwayi. |  |  |  |  |  |
| Gushyira amajwi yafashwe muri sisitemu y’amakuru byari byoroshye kandi bikoreka. |  |  |  |  |  |
| Ifatwa ry’aya amajwi byagize ingaruka ku buryo nasuzumyemo abarwayi. |  |  |  |  |  |
| Nsanga amahugurwa cyangwa inama byiyongera ku gukoresha imfatamajwi mu masuzuma yaba ingirakamaro |  |  |  |  |  |

##

##

##

##

##

##

##

##

##

##

##

##

##

##

##

##

## **Information and Written Consent Form - CHW - English**

**Project Title**: *Assessing the Potential Utility of Large Language Models for Assisting Community Health care Workers in Rwanda*

**Version Date**: February 18, 2025

**Principal Investigator**: Dr. Natnael Shimelash

**Hello!** Thank you for considering participation in this study. We are conducting research to understand how technology can support Community Health Workers (CHWs) in providing better care to patients. To achieve this, we need to record CHW-patient interactions and run it through our system to train the system and test its performance and value in healthcare. Your role in this study is crucial.

Your role in this study

- You will obtain informed consent from patients. This ensures that patients are fully aware of the purpose of the study and their role in it before any recordings are made.
- Once patients provide their consent, you will conduct your consultations as you usually would, without changing your approach or style. After each consultation you will ask your patient a set of questions to assess the patient's experience being recorded for this study.
- After, we also need you to conduct regular 5 to 14-day visits to patients in this study and collect counter referrals for those referred.
- At the end of the study we will give you a survey and do a group discussion to learn about your experience using the Application.

This data collection period will involve approximately 100 CHWs across Rwanda and is expected to last about one month. By agreeing to participate, you will allow us to record your interactions with patients during consultations, conduct regular 5 to 14-day visits to patients in this study, and collect counter referrals. These recordings will help researchers understand how our technology system can enhance the work of CHWs and improve the quality of patient care.

**Participation is voluntary.**

It is your choice whether to take part in this study or not. Should you decide to take part, you have the right to change your mind and leave the study at any time. Declining to participate or halting your participation are within your full rights and will not condemn you to any consequence.

**Important Information About Your Participation**

- **No Judgement or Evaluation**: This study is not about evaluating your performance. We are not recording your consultations to grade or evaluate your service in any way. We want you to conduct your consultations as if the study were not happening—just as you normally do.
- **Privacy and Confidentiality**:
- Your identity and personal information will remain private. The recordings will not include your name or any other identifiable information.
- Each participant will be assigned a unique participant identity number (PIN).
- The recordings will be securely stored on password-protected servers accessible only to the research team.
- None of the recordings will be shared with anyone in a way that can identify you. All data used in reports or publications will be aggregated and de-identified to protect your privacy.
- **Use of Data**: The recordings will only be used to design technologies that better support CHWs. The information will be analyzed collectively, focusing on overall patterns and insights, rather than on individual participants.

**What are the possible risks or discomforts related to taking part in this project?**

The additional steps to collect informed consent and conduct follow-ups may increase your workload. This will be taken into consideration to compensate you for the additional work.

If you have been invited to travel for this study, we will consider your transport expenditure and financial reimbursements will be provided at the end of the study. Please note that deciding to opt out of the study will not prevent you from getting your transport fee reimbursements.

**Potential Benefits**

Your participation will contribute to the development of tools that can improve health care delivery for both CHWs and patients. The results of this study will be shared with the government of Rwanda to guide improvements in CHW programs and may also be published in scientific journals to inform broader research.

**Will I be compensated for participating in this research?**

We acknowledge that participating in this study can increase your workload. You will be compensated a flat rate of 4,000 Rwf per day for the duration of the study.

**What You Need to Know**

- We will provide a two-day training to explain the study to patients, collect their informed consent, and enroll them as participants.
- Participation is completely voluntary. You can choose to say yes or no without any consequences for your work as a CHW.
- You can withdraw your participation at any time

**If you have any questions, concerns, or complaints about this project, you can talk to the researchers for this study** Cyprien Nshimiyimana who can be reached at cnshimiyimana@c4ir.rw, and on mobile at 0781474375 if, you have questions, concerns, or complaints.

This research study has been reviewed by the Rwandan National Ethic Committee (RNEC). If any of the scenarios below apply to you, please contact the RNEC Chairperson, Dr. Vedaste Ndahindwa, at **** *** *** *** or the RNEC Secretary, Dr. Marie Francoise Mukanyangezi, at ************.

- If you have any ethical concerns regarding the research or research team;
- If you have unanswered questions, or concerns, by the research team;
- If you cannot reach the research team;
- If you have questions about your rights as a research participant, or;
- If you think the project has harmed you.

**Do you have any questions for me about this study, or would you like me to clarify anything?**

**Are you willing to participate in this study, including recording your consultations with patients (who have provided their consent) for the purposes of this research?**

| Full name and signature of consenter | Full name and signature of witness | Full name and signature of person asking for consent |
| --- | --- | --- |

Date and location: _______________________

## **Information and Written Consent Form - CHW - Kinyarwanda**

AMAKURU N’IFISHI Y’UBURENGANZIRA BUTANZWE MU MAGAMBO IGENEWE UMUJYANAMA W’UBUZIMA

**Umutwe w’umushinga**: Isuzumabushobozi rya Porogaramu za Mudasobwa Zifashisha Amakuru Menshi y’Ururimi Zabitswemo mu gufahsa bajyanama b’ubuzima mu Rwanda

**Itariki ya Verisiyo:** February 18, 2025

**Ukuriye ubushakashatsi:** Dr. Natnael Shimelash

**Muraho neza!** Murakoze gutanga umwanya wanyu muri ubu bushakashatsi. Turi gukora ubushakashatsi bugamije gusobanukirwa uruhare ikoranabuhanga ryakoreshwa mu gufasha abajyanama b’ubuzima guha abarwayi serivisi z’ubuzima zinoze. Kugira ngo ibi bigerweho, turifuza gufata mu majwi ibiganiro umujyanama w’ubuzima agirana n’umurwayi, hanyuma tukabiha sisitemu z’ikoranabuhanga zacu za mudasobwa kugirango tuyigishe tunasobanukirwe ubushobozi n’imikorere byayo. Uruhare rwawe muri ubu bushakashatsi ni ingenzi cyane

Uruhare rwawe muri ubu bushakashatsi

- Abarwayi bazabanza gutanga icyemeza ko bemera kugira uruhare ku bushake. Ibi byemezako abarwayi basobanukiwe intego y’ubushakashatsi n’uruhare rwabo muri bwo mbere yo gufatwa amajwi.
- Mu gihe umurwayi yemeje kugira uruhare, uzakora isuzuma ryawe nk’ibisanzwe udahinduye imyitwarire n’uburyo usanzwe uzikoramo. Nyuma ya buri suzuma uzajya ubaza umurwayi ibibazo kugirango umenye uko bakiriye gufatwa amajwi
- Nyuma y’ibyo, dusabwe gusura umurwayi mu buryo buhoraho mu minsi iri hagati y’itanu na 14 gukusanya no kureba impinduka y’ibyemejwe.
- Kumusozo w’ubu bushakashatsi tuzakusanya amakuru yerekeye uko mwakiriye iri koranabuhanga.

Biteganijweko abajyanama b’ubuzima bagera ku 100 bo hirya no hino mu gihugu aribo bazitabira ubu bushakashatsi buzamara Ukwezi kumwe. Kwemera kugira uruhare, uzaba wemeye kujya ufatwa amajwi y’ibiganiro ugirana n’abarwayi mu isuzuma, gusura umurwayi uri muri ubu bushakashatsi bihoraho mu gihe kiri hagati y’imisi 5 na 14, ukamenya uko agenda amererwa. Aya majwi azafasha abashakashatsi gusobanukirwa uko ikoranabuhanga rya kwifashishwa mu kunoza imikorere y’abajyanama b’ubuzima no kuzamura urwego rwo kwita k’umurwayi.

**Kwitabira ni kubushake**

Ni amahitamo yawe kugira cyangwa kutagira uruhare muri ubu bushakashatsi. Mu gihe wemeye kugira uruhare muri ubu bushakashatsi, ufite uburenganzira bwo guhindura intekerezo ukabivamo igihe icyo aricyo cyose. Mu gihe utemeye cyangwa ushidikanya kugira uruhare muri ubu bushakashatsi ntangaruka mbi bizakugiraho.

**Amakuru y’ingenzi ukwiriye kumenya nk’ufite uruhare mu bushakashatsi.**

- **Ntabwo ari isuzumwa**: ubu bushakashatsi ntibugamije gupima imikorere yawe. Ntabwo gufata amajwi yawe mu gihe usuzuma umurwayi bigamije ku gupima, kukujora, cyangwa kuguhana mu buryo ubwo aribwo bwose. Turagusaba gukora amasuzuma yawe nkaho ubu bushakashatsi budahari- bikore nkuko usanzwe ubigenza.
- **Ibanga ry’amakuru**
  - Umwirondoro wawe n’amakuru yawe bizagirwa ibanga. Amajwi ufatwa ntazagaragaza umwirondoro wawe.
  - Buri mujyanama w’ubuzima azahabwa umubare bwite w’ibanga.
  - Amajwi turafata azabikwa muri za mudasobwa zirimo ijambobanga riyarinze cyane, azagerwaho gusa n’abagize itsinda ry’ubwo bushakashatsi.
  - Amajwi yawe yose azifashishwa mu gutangaza ibyabonetse azakurwaho umwirondoro mu ku kurinda kumenyekana. Ntamajwi yawe n’amwe azifashishwa mu buryo bugutangaza
- **Imikoreshereze y’amakuru**: amajwi yafashwe azifashishwa gusa mu kubaka ikoranabuhanga ryunganira abajyanama b’ubuzima. Amakuru azasesengurwa mu buryo bwa rusange, harebwa imiterere rusange yayo kuruta uruhare rw’umuntu ku giti cye.

**Ni izihe mu ngaruka cyangwa imbogamizi zifitanye isano no kugira uruhari muri ubu bushakashatsi?**

Intambwe z’inyongera zo gukusanya uburenganzira bushingiye ku kumenyeshwa neza no gukora ubugenzuzi bushya bishobora kongera imirimo mufite. Ibi bizitabwaho kandi hazateganywa impinduramatwara y’inyongera kugira ngo haboneke impamvu z’igihembo cy’iyo mirimo y’inyongera.

Niba mwatumiwe kugira ingendo muri ubu bushakashatsi, tuzita ku mafaranga y’ingendo mukoresha, kandi amafaranga yo kwishyura ingendo azatangwa nyuma y’isozwa ry’ubushakashatsi. Icyakora, mwamenya ko gufata icyemezo cyo kuva muri ubu bushakashatsi bitazababuza guhabwa amafaranga y’ingendo mwemerewe.

**Inyungu zitezwe**

Amakuru yawe azagira uruhare mu kubaka sisitemu zifasha abajyanama b’ubuzima n’abarwayi kubona no gutanga serivisi z’ubuzima nziza. Amakuru azava muri ubu bushakashatsi azahabwa Leta y’u Rwanda mu gukomeza kunoza serivisi z’abajyanama b’ubuzima, ndetse ashobora no kuzatangazwa mu mbunga ntangazabushakashatsi za siyansi kugira ngo hakorwe ubushakashatsi bwagutse.

**Ese hari insimburamibyizi nzahabwa ku bwo kugira uruhare muri ubu bushakashatsi?**

Turabizi ko kwitabira ubu bushakashatsi bishobora kongera imirimo mufite. Muzahabwa insimburamubyizi ingana na 4,000 Rwf ku munsi mu gihe cyose ubushakashatsi buzamara.

**Ibyo ukwiye kumenya**

- Abarwayi bazahugurwa ku bijyanye n’ubu bushakashatsi, buzuze amasezerano yo kugira uruhare, hanyuma bandikwe mu bazagira uruhare mu bushakashatsi.
- Kugira uruhare ni ubushake. Wahitamo kuvuga yego cyangwa oya kandi ntibizakugiraho ingaruka kukazi kawe nk’umujyanama w’ubuzima.
- Wemerewe kwikura mu bushakashatsi igihe cyose ubishakiye.

**Ndamutse ngize ikibazo cyangwa imbogamizi kuri ubu bushakashatsi wakwitabaza umushakashatsi** ugize iryo tsinda NSHIMIYIMANA Cyprien uboneka kuri imeli [cnshimiyimana@c4air.rw](mailto:cnshimiyimana@c4air.rw), na telefone 0781474375 mu gihe ufite ikibazo, icyifuzo, imbogamizi, cyangwa hari ibyo utishimiye.

Ubu bushakashatsi bwagenzuwe na Komite y'Igihugu ishinzwe imyitwarire iboneye mu bushakashatsi (RNEC). Niba kimwe muri ibi bikorwa bikurikira kikubayeho, nyabuna, hamagara umuyobozi wa Komite ya RNEC, Dr. Vedaste Ndahindwa, kuri **** *** *** *** cyangwa Umunyamabanga wa RNEC, Dr. Marie Francoise Mukanyangezi, kuri ************.

● Niba mufite impungenge zijyanye n'imyitwarire iboneye muri ubu bushakashatsi cyangwa itsinda ry'abashakashatsi;

● Niba mufite ibibazo bitarabonerwa ibisubizo cyangwa impungenge mutarakemurirwa n'itsinda ry'ubushakashatsi;

● Niba mudashobora kugera ku itsinda ry'ubushakashatsi

● Niba mufite ibibazo bijyanye n'uburenganzira bwanyu nk'abitabiriye ubushakashatsi, cyangwa; Niba mubona ko uyu mushinga wabagizeho ingaruka.

**Hari ikibazo cyerekeye ubu bushakashatsi ukeneye kumbaza? Cyangwa kugira icyo usobanukirwa?**

**Urifuza kugira uruhare muri ubu bushakashatsi, gufatwa amajwi usuzuma abarwayi (bemeye kugira uruhare muri ubu bushakashatsi) ku nyungu z’ubu bushakashatsi?**

| Amazina n’umukono by’uwemeye kugira uruhare | Amazina n’umukono y’umutangabuhamya | Amazina n’umukono y’ufata amasezerano yo kwitabira ku bushake |
| --- | --- | --- |

Ahantu n'itariki.: _______________________

**Information and Written Consent Form - Patient - English**

**Project title:** Assessing the Potential Utility of Large Language Models for Assisting Community Health care Workers in Rwanda

**Version date:** May 23, 2025

**Principal Investigator:** Dr. Natnael Shimelash

**Hello!** Before our consultation today, I need to explain something important and get your permission. I am taking part in a study to help make health care better in our community by using technology. For this study, I would need to record our conversation today. This recording will help researchers understand how to support us as community health workers to serve you better.

This study will involve approximately 100 CHWs and 800 patients in Rwanda and is expected to last about two months. By agreeing to participate, you will allow me to record our interaction today, and to visit you in 5 and 14 days from today to follow up on your progress and collect any relevant counter referrals from health centers if applicable. This information will be used to inform the programmers what they should add/remove or improve when they make the AI technology that helps Community Health care Workers in Rwanda. At the end of the consultation, I will ask you some additional questions about your experience being recorded in the study.

**Participation is voluntary.**

It is your choice whether to take part in this study or not. Should you decide to take part, you have the right to change your mind and leave the study at any time. If you agree to record our session today, there is no additional procedure. I will give you service as usual. Declining to participate or halting your participation will not condemn you to any consequences. Whether you participate or not, I will serve you as normal.

**Privacy and Confidentiality**:

Your identity and personal information will remain private. The recordings will not include your name or any other identifiable information. Instead, you will be assigned a unique participant identity number (PIN). The recordings will be securely stored on password-protected servers accessible only to the research team. None of the recordings will be shared with anyone in a way that can identify you. All data used in reports or publications will be aggregated and de-identified to protect your privacy.

**Use of Data**:

The recordings will only be used to design technologies that better support CHWs. The information will be analyzed collectively, focusing on overall patterns and insights, rather than on individual participants.

**Possible risks or discomforts related to taking part in this project?**

We do not expect any direct risks to your health by taking part in this study. However, there may be minimal risks of data breach which we have accounted for by de-identifying your personal information using PINs, limiting access to data to researchers, and storing the information in secure servers.

**Potential Benefits**

Your participation will contribute to the development of tools that can improve health care delivery for both CHWs and patients. The results of this study will be shared with the government of Rwanda to guide improvements in CHW programs and may also be published in scientific journals to inform broader research.

**Here are the most important things for you to know:**

- **Participation is voluntary.** You can say yes or no to be included in this study.
- There are no consequences if you say no, you will still get the same care from me as always
- You can ask me to stop recording at any time during our talk or decline a follow-up visit at any time without any implications.
- If you decide to stop your participation in the study midway we will delete entirely the data collected before your decision.
- There is no compensation for participating in this research study.

**If you have any questions, concerns, or complaints about this project, you can talk to the researchers for this study** Cyprien Nshimiyimana who can be reached at cnshimiyimana@c4ir.rw, and on mobile at 0781474375 if, you have questions, concerns, or complaints.

This research study has been reviewed by the Rwandan National Ethic Committee (RNEC). If any of the scenarios below apply to you, please contact the RNEC Chairperson, Dr. Vedaste Ndahindwa, at **** *** *** *** or the RNEC Secretary, Dr. Marie Francoise Mukanyangezi, at **** *** *** ***.

- If you have any ethical concerns regarding the research or research team;
- If you have unanswered questions, or concerns, by the research team;
- If you cannot reach the research team;
- If you have questions about your rights as a research participant, or;
- If you think the project has harmed you.

**Do you have any questions for me about this study, or would you like me to explain anything again?**

**Are you willing to let me record our consultation today for the study?**

| Full name and signature of consenter | Full name and signature of witness | Full name and signature of person asking for consent |
| --- | --- | --- |

Date and location: _______________________

##

## **Information and Written Consent Form - Patient - Kinyarwanda**

AMAKURU N’ UBURENGANZIRA BUTANZWE MU MAGAMBO - Umurwayi

**Umutwe w’umushinga:** Umutwe w’umushinga: Isesengura ry’Akamaro ka Mudasobwa Zifashisha Amakuru Menshi y’Ururimi Zabitswemo (LLM) zaba zifite mu gufasha abajyanama b’ubuzima mu Rwanda

**Itariki ya verisiyo :** February 18, 2025

**Ukuriye ubushakashatsi:** Dr. Natnael Shimelash

**Muraho!** Murakoze guhitamo kuba mwagira uruhare muri ubu bushakashatsi. Turimo gukora ubushakashatsi kugira ngo dusobanukirwe uko ikoranabuhanga ryafasha Abajyanama b’Ubuzima (CHWs) mu kurushaho kwita ku barwayi. Kugira ngo tubigereho, turifuza gufata amajwi y'ibiganiro hagati y'Umujyanama w’Ubuzima n’Umurwayi, hanyuma tukayakoresha muri sisitemu yacu mu kwigisha no gusuzuma imikorere n’agaciro k’iri koranabuhanga muri serivise z’ubuzima. Uruhare rwawe muri ubu bushakashatsi ni ingenzi cyane.

Ubu bushakashatsi turimo buzakoresha Abajyanama b’Ubuzima bagera kuri 100 n’abarwayi 800 mu Rwanda, kandi biteganyijwe ko buzamara amezi abiri. Niwemera kubyitabira, uzemera ko mfata amajwi y’ibiganiro byacu by’uyu munsi, kandi nkazagusura nyuma y’iminsi 5 na 14 kugira ngo nkukurikirane no kugirango menye amakuru ya taransiferi wahawe n’ikigo nderabuzima, niba biri ngombwa. Aya makuru azafasha abashakashatsi gusobanukirwa uko ikoranabuhanga ryacu rishobora gufasha abajyanama b’ubuzima mu Rwanda. Kumpera Y’isuzuma, nzakubaza ibindi bibazo by’inyongera kubigendanye n’uko wakiriye gufatwa amajwi muri ubu bushakashatsi.

**Kwitabira ni k’ubushake**

Ni amahitamo yawe kugira cyangwa kutagira uruhare muri ubu bushakashatsi. Mu gihe wemeye kugira uruhare muri ubu bushakashatsi, ufite uburenganzira bwo guhindura intekerezo ukabivamo igihe icyo aricyo cyose. Mu gihe utemeye cyangwa ushidikanya kugira uruhare muri ubu bushakashatsi ntangaruka mbi bizakugiraho.

**Ibanga ry’amakuru**

Amakuru yawe bwite n'ibiranga umwirondoro wawe bizaguma ari ibanga. Amajwi azakusanywa ntabwo azaba arimo amazina yawe cyangwa ikindi cyose cyatuma umenyekana. Ahubwo, uzahabwa numero yihariye (PIN) izakuranga nk'uwitabiriye ubushakashatsi. Amajwi azabikwa ahantu hatekanye, muri seriveri zifite umubare w'ibanga (password) zizajya zigerwaho gusa n'itsinda ry'abashakashatsi. Nta na hamwe amajwi azatangarizwa ku buryo wamenyekana. Amakuru yose azakoreshwa mu bitabo by'ubushakashatsi azaba akenwe kandi atagaragaza umwirondoro w'umuntu kugira ngo turinde ubuzima bwawe bwite

**Zimwe mu ngaruka cyangwa imbogamizi zifitanye isano no kugira uruharin muri ubu bushakashatsi**

Nta ngaruka z’ako kanya twumva uzahura nazo ni ugira uruhare muri ubu bushakashatsi. Icyakora, hashobora kubaho ingaruka ntoya zo kugaragara k’umwirondoro wawe zishingiye mu ifungurwa ry’amakuru watanze hakoreshejwe ijambo banga rikuranga, kugabanya uburyo bwo kubona makuru ku bashakashatsi no kubika amakuru mu bubiko butekanye.

**Inyungu zitezwe**

Amakuru yawe azagira uruhare mukubaka sisitemu zifasha abajyanama b’ubuzima n’abarwayi kubona no gutanga serivisi z’ubuzima nziza. Amakuru azava muri ubu bushakashatsi azahabwa Leta y’u Rwanda mu gukomeza kunoza serivisi z’abajyanama b’ubuzima, ndetse ashobora no kuzatangazwa mu mbunga ntangazabushakashatsi za siyansi kugira ngo hakorwe ubushakashatsi bwagutse.

**Dore ibintu by'ingenzi ugomba kumenya:**

- **Kwitabira ni k’ubushake.** Ushobora kwemera cyangwa guhakana kugira uruhare muri ubu bushakashatsi.
- Nta ngaruka mbi uzagira mu gihe uvuze Oya mu gihe icyo ari cyo close cy'isuzumwa ryawe, uzakomeza uhabwe ubufasha bukwiriye nk'uko usanzwe ubuhabwa
- Mu gihe uhisemo guhagarika uruhare mo hagati uruhare rwawe muri ubu bushakashatsi tuzahita dusiba burundu amakuru yakusanyijwe mbere y'uko ufata icyo cyemezo.
- Nta gihembo gihabwa uwagize uruhare muri ubu bushakashatsi.

**Ndamutse ngize ikibazo cyangwa imbogamizi kuri ubu bushakashatsi wakwitabaza umushakashatsi** ugize iryo tsinda NSHIMIYIMANA Cyprien uboneka kuri imeli [cnshimiyimana@c4air.rw](mailto:cnshimiyimana@c4air.rw), na telefone 0781474375 mu gihe ufite ikibazo, icyifuzo, imbogamizi, cyangwa hari ibyo utishimiye.

Ubu bushakashatsi bwagenzuwe na Komite y'Igihugu ishinzwe imyitwarire iboneye mu bushakashatsi (RNEC). Niba kimwe muri ibi bikorwa bikurikira kikubayeho, nyabuna, hamagara umuyobozi wa Komite ya RNEC, Dr. Vedaste Ndahindwa, kuri **** *** *** *** cyangwa Umunyamabanga wa RNEC, Dr. Marie Francoise Mukanyangezi, kuri **** *** *** ***.

● Niba mufite impungenge zijyanye n'imyitwarire iboneye muri ubu bushakashatsi cyangwa itsinda ry'abashakashatsi;

● Niba mufite ibibazo bitarabonerwa ibisubizo cyangwa impungenge mutarakemurirwa n'itsinda ry'ubushakashatsi;

● Niba mudashobora kugera ku itsinda ry'ubushakashatsi

● Niba mufite ibibazo bijyanye n'uburenganzira bwanyu nk'abitabiriye ubushakashatsi, cyangwa; Niba mubona ko uyu mushinga wabagizeho ingaruka.

**Hari ibibazo wambaza bijyanye n’iri fatwa ry’amajwi ? cyangwa urashaka ko hari icyo nongera kugusobanurira?**

**Urumva wanyemerera ngafata amajwi mu gihe ndi kugusuzuma kuri uyu munsi?**

| Amazina n’umukono by’uwemeye kugira uruhare | Amazina n’umukono y’umutangabuhamya | Amazina n’umukono y’ufata amasezerano yo kwitabira ku bushake |
| --- | --- | --- |

Ahantu n'itariki.: _______________________

**Ese waba wemeye ko nzagusura nyuma y’iminsi 5 na 14 kugira ngo nkukurikirane no kugirango menye amakuru ya taransiferi wahawe n’ikigo nderabuzima, niba biri ngombwa?**

| Amazina n’umukono by’uwemeye kugira uruhare | Amazina n’umukono y’umutangabuhamya | Amazina n’umukono y’ufata amasezerano yo kwitabira ku bushake |
| --- | --- | --- |

Ahantu n’itariki: _______________________
